# Effect of KI Concentration in Correcting Tank on Optical Properties of PVA Polarizing Film

**DOI:** 10.3390/polym14071413

**Published:** 2022-03-30

**Authors:** Yang Yang, Ziyuan Zheng, Jun Lin, Lintao Zhou, Guohua Chen

**Affiliations:** College of Materials Science and Engineering, Huaqiao University, Xiamen 361021, China; Y18959265136@163.com (Y.Y.); 20013081064@stu.hqu.edu.cn (Z.Z.); 19014087012@stu.hqu.edu.cn (J.L.); 20014087051@stu.hqu.edu.cn (L.Z.)

**Keywords:** PVA polarizing film, correction process, transmittance, chromaticity, degree of polarization

## Abstract

Polarizer is a key component of the liquid crystal display panel, and the optical properties mainly include transmittance, degree of polarization and chromaticity. Polarizer is made of a multilayer optical film, in which the PVA polarizing film is the core structure for realizing the polarization of the whole polarizer. PVA polarizing film is commonly manufactured through a multi-step craft, including rinsing, dyeing, stretching and correcting. The correction process has a significant impact on the final apparent color and optical properties of the polarizer. In this study, the KI concentration in the correcting tank, ranging from 1% to 3%, was systematically investigated. With the increase in KI concentration, the Raman vibration peak at 160 cm^−1^ representing $I_{5}^{-}$ ions gradually weakened, while the Raman vibration peak at 110 cm^−1^ representing $I_{3}^{-}$ ions gradually increased, indicating that the KI in the correcting tank changed the chemical equilibrium of polyiodide ions in PVA. Then abundant chromophore $I_{5}^{-}$ ions were consumed and $I_{3}^{-}$ ions generated, so that the apparent color of PVA polarizing film gradually changed from dark blue to dark gray, and the chromaticity *a*-value and *b*-value gradually increased. The change in the concentration of dichroic species ($I_{5}^{-}$ and $I_{3}^{-}$) in PVA polarizing film had directly affected its transmittance in the visible range. From the UV-Vis transmittance spectrum of PVA polarizing film, when the $I_{5}^{-}$ ions were consumed and $I_{3}^{-}$ ions generated, the transmittance of PVA polarizing film in the region of 675–525 nm wavelength increased gradually while the polarization degree also increased.

## 1. Introduction

Among the many new flat panel display technologies, thin film transistor liquid crystal display (TFT-LCD) occupies a dominant position in the market due to its numerous advantages, such as lightness and thinness, low working voltage, low power consumption, low electromagnetic radiation, high resolution, and large visible area [1]. Polarizer is the key structure to liquid crystal display. In the whole liquid crystal module, two polarizers form a polarized optical system together. The liquid crystal with optical and electrical anisotropy, combined with the thin film transistor (TFT), plays a switching role for the electrical signal driving liquid crystal to modulate the light incident into the liquid crystal cell by backlight [2,3]. In many types of polarizers, iodine polarizers are most widely used due to their advantages of high transmittance and high degree of polarization [4].

Poly (vinyl alcohol, PVA) as a widely used material, in recent years, often was combined with various nanoparticles to form composites to improve its physicochemical properties and expand its application fields [5,6,7,8]. Besides, PVA is one of the most widely used raw materials for the polarizer, which realizes polarization by one-dimensionally aligning dichroic polyiodine absorbed in PVA membrane [9,10]. The blue color reaction of PVA and iodine was observed by Herrmann, who first synthesized PVA, and Staudinger et al. as early as 1927 [11,12]. Since then, the PVA–iodine complex has aroused the interest of many researchers, and a large number of related research has been carried out. Originally, some scholars often compared blue PVA–iodine complex with the blue amylose–iodine complex [13,14] and inferred the conformation of the PVA chain segment in the complex. For example, Zwick [15] suggested that a linear polyiodine was helically surrounded by a PVA chain, and this idea was later supported by Inagaki et al. [16] on the basis of the similarity of Raman spectroscopy between PVA–iodine and amylose–iodine complexes. However, Rundle et al. [17] and Tebelev et al. [18] suggested that a linear polyiodine might be surrounded by several PVA chains. Miyasaka [10] proposed a model of the $I_{5}^{-}$ complex formed in the PVA amorphous phase, in which the structure had four syndiotactic PVA segments with the extended conformation surrounding a polyiodine. Recently, Tashiro et al. [19] proved that the vertically arrayed linear $I_{5}^{-}$ ions were surrounded by six PVA chains of planar-zigzag conformation to form the cylindrical structure when the PVA sample was immersed in an iodine solution with an extremely low concentration of 0.001~0.05 M.

The optical properties of PVA polarizing film are closely related to the PVA–iodine complex, which can produce the dichroic species and show the maximal absorption peak in the frequency region of visible-ray wavelength. There are many factors affecting the conformation of the PVA–iodine complex, such as the degree of polymerization of PVA molecules [20,21], I_2_, KI or H_3_BO_3_ concentration [22,23,24,25,26,27], draw ratio [28,29], and stereoregularity of PVA molecules [30,31,32,33,34]. Subsequent studies indicated that polyiodines participating in the PVA–iodine complex mainly contained $I_{3}^{-}$ and $I_{5}^{-}$. Some studies [16,35,36,37,38] revealed the iodine ion species in the PVA–iodine complex by resonance Raman spectra. The Raman bands could be detected at about 160 cm^−1^ and 110 cm^−1^ due to the vibrations of $I_{3}^{-}$ and $I_{5}^{-}$ ion species. Mokhnach and Zueva [39] studied iodine–PVA in an aqueous solution and then proved that in the complex there were four absorption peaks at 226, 288, 350 and 620 nm. When the complex was heated, the blue disappeared; in addition, only the 226, 288 and 350 nm peaks still remained. Subsequently, Zwick [15] reported that the PVA–iodine blue complex showed four absorption peaks at 226, 290, 355 and 650 nm, which were assigned to $I^{-}$, $I_{3}^{-}$, $I_{3}^{-}$ and $I_{5}^{-}$ respectively, and the last peak was considered to belong to the chromophric polyiodide ion. Oishi and Miyasaka [40] and Hayashi et al. [41] proposed that PVA formed a complex with iodine in the amorphous region when PVA films were soaked in comparatively lower concentrated iodine/KI solutions. Choi et al. [42] reported that iodine intruded into PVA crystals and formed co-crystals with PVA as the iodine concentration in the soaking solution was above 2 × 10^−2^ mol L^−1^. Sakuramachi et al. [43] first reported that the absorption spectra of PVA films soaked at very high iodine concentrations (higher than 2 × 10^−2^ mol L^−1^) was about 470 nm.

Boric acid and the syndiotactic degree of PVA are also significant to the color development of the complex. Takamiya et al. [44] came to the conclusion that isotactic PVA showed no color development but an increase in syndiotacticity-rich PVA film. Meanwhile, lower temperatures, the elongation of complex film, and the presence of boric acid enhanced the absorbance at 600 nm due to $I_{5}^{-}$. Woo et al. [45] proposed that boric acid formed intra-molecular cross-links on the PVA chain to accelerate the formation of the PVA–iodine complex.

Up to now, the preparation of PVA polarizing film still follows the principle of “H-sheet” invented by Edwin H. Land, founder of the American Polaroid company in the 1930s [46]. The chemical reaction mechanism is the same as that of the PVA-I-H_3_BO_3_ reaction system. Although a large number of PVA-I-H_3_BO_3_ reactions have been studied before, as summarized above, the preparation process of PVA polarizing film has not been reported in detail. So, detailed information on its process is worthy of being further revealed.

The preparation process of PVA polarizing film mainly includes rinsing, dyeing, stretching and correcting steps. The correction process plays a significant role in the final optical properties and apparent color of PVA polarizing film. The KI in the correcting tank is mainly used to regulate the concentration of dichroic species in PVA polarizing film. Various concentrations of dichroic species cause different optical properties of the PVA polarizing film. Therefore, this paper mainly discusses the effect of KI concentration in a correcting tank on optical properties of PVA polarizing film and proposed related mechanism. In the present study, the transmittance, degree of polarization, chromaticity and apparent color of the PVA polarizing film are objectively recorded when the KI concentration in the correcting tank ranges from 1% to 3%. Such research records not only fill the vacancy of the detailed preparation process and mechanism of the PVA polarizing film, but also have important guiding significance for the practical production of the polarizer.

## 2. Materials and Methods

### 2.1. Materials

Polyvinyl alcohol (PVA), with a polymerization degree of 1700, alcoholysis degree of over 98% and saponification degree of over 99%, was purchased from Aladdin Biochemical Technology Co., Ltd. (Shanghai, China). Methanol (purity ≥ 99.5%), glycerol (purity ≥ 99.0%), iodine (I_2_, purity ≥ 99.8%), potassium iodide (KI, purity ≥ 99.0%), boric acid (H_3_BO_3_, purity ≥ 99.5%) and zinc chloride (ZnCl_2_, purity ≥ 98.0%) were procured from Sinopharm Chemical Reagent Co., Ltd. (Shanghai, China). All solvents used for the reactions were of analytical grade. Ultrapure water was used in the whole experiments.

### 2.2. Preparation of PVA Membrane

To start with, 12% PVA powder, 10% methanol and 2% glycerol were put into a beaker containing 150 g ultrapure water, swelled at 40 °C for 1 h, and then heated up to 80 °C for dissolution. After standing and defoaming, a given amount of solution was poured on the clean mirror stainless steel plate. The PVA liquid coating was applied to the plate with a film scraper, and then baked in a vacuum-drying oven at 40 °C for 6 h, followed by 70 °C for 3 h to obtain a 80-μm-thick PVA film. Finally, the obtained PVA film was cut into 6 cm × 8 cm rectangular films for use in this experiment.

### 2.3. Preparation of PVA Polarizing Film

**Figure 1:**
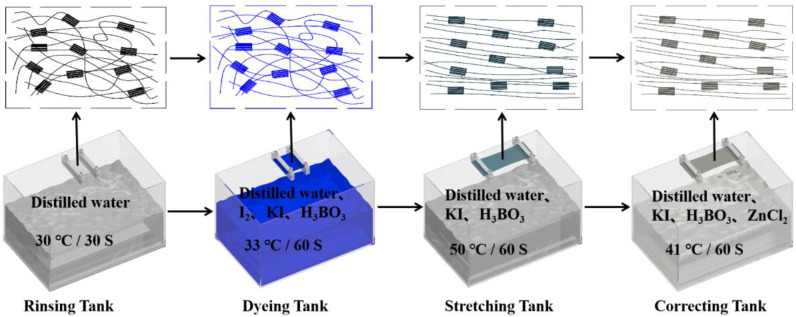
Preparation process of PVA polarizing film.

As mentioned above, the PVA polarizing film was mainly prepared through rinsing, dyeing, stretching and correcting. The preparation process is shown in Figure 1, and the specific steps were as follows: (1) The PVA original membrane was clamped on the self-made stretching machine, and then soaked in the rinsing tank at 30 °C for 30 s. On the one hand, the rinsing process could wash away impurities on the surface of PVA original membrane, such as the plasticizer. On the other hand, it could make PVA absorb water, swell and reduce the crystallization area, which is conducive to the entry of iodine ions into PVA, as Song et al. reported [47]; (2) The rinsed PVA membrane was moved to a 33 °C dyeing tank for 60 s, wherein the PVA membrane could absorb the dichroic iodine ions and cross-link with H_3_BO_3_ to form the $I_{5}^{-}$ ion chromophore. The PVA membrane turned dark blue during this progress; (3) After dyeing, the iodine ions were arranged in a disorderly manner in the curled PVA molecular chain, as shown in Figure 1 (dyeing tank). In order to get the polarized iodine ions, the dyed PVA film was soaked in a 50 °C stretching tank and stretched to the required draw ratios. Extension can make the iodine ions orderly arranged along the stretching direction to form a long chain. The stretched PVA film could absorb polarized light parallel to its arrangement direction, which meant that only polarized light perpendicular to the arrangement direction was allowed to pass through. In addition, the KI in the stretching tank could change the chemical correction of iodine ions in PVA and urge iodine ions to form $I_{3}^{-}$ and $I_{3}^{-}\cdot I_{2}$; (4) The stretched PVA film then was placed in a correcting tank at 41 °C for 60 s. KI in the correcting tank can change the chemical balance of polyiodide ions in PVA, generate $I_{3}^{-}$, and finally adjust the properties of PVA polarizing film by changing the concentration of KI; (5) The structure of the PVA polarizing film prepared through the steps above must also be fixed by the drying process. The drying process was not only to solidify the PVA polarizing film, but also to improve the color fixing reaction. Therefore, the PVA polarizing film was placed in a vacuum drying oven for 10 min at 50 °C and 10 min at 70 °C for gradient drying. Besides ensuring the fixation of PVA structure, it also ensured the smooth progress of the color-fixation reaction. With regard to the solution composition in each tank in the process above, distilled water was used in the rinsing tank, and the proportions of liquid medicine contained in the dyeing, stretching and correcting tanks are shown in Table 1.

### 2.4. Characterization

Samples were characterized by the color-difference meter, micro-confocal Raman spectrometer, ultraviolet-visible spectrophotometer (UV-Vis) and Sony camera. After the apparent color of the sample was recorded by the camera, the chromaticity—including *a*-value and *b*-value—were measured by the ZE6000 color-difference meter(Electric Color Co., Ltd., Tokyo, Japan). Raman spectroscopy was taken with a He-Ne laser (532 nm) as the excitation source using an InVi/InVia micro-confocal Raman spectrometer(Renishaw company, London, England). The transmittance spectrum of PVA polarizing films were measured with the ultraviolet-visible spectrophotometer, which was based on the Thermo Evolution 201 model (Thermo Fisher Technology Co., Ltd., New York, NY, USA), with a test accuracy of 1nm and test wavelength in the range of 400~700 nm. Subsequently, the single transmittance ($T_{s}$), parallel transmittance ($T_{∥}$), perpendicular transmittance ($T_{⊥}$), and degree of polarization of the PVA polarizing film were calculated. Particularly noteworthy was the fact that $T_{∥}$ meant the transmittance of two mutually superimposed PVA polarizing films whose light absorption axes were parallel to each other, while $T_{⊥}$ meant perpendicular.

## 3. Results and Discussion

### 3.1. Characterization and Analysis of Chromaticity

**Figure 2:**
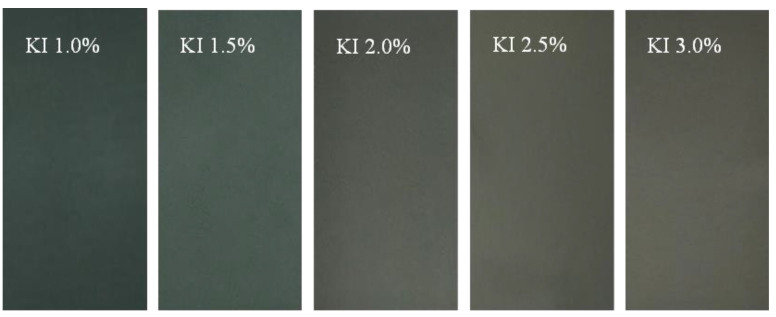
The comparison of PVA polarizing films which were stretched 6 times prepared in the correcting tank with the concentration of KI from left to right being set to 1.0%, 1.5%, 2.0%, 2.5% and 3.0%, respectively.

In order to explore the effects of KI concentration in the correcting tank on PVA polarizing film, a series of PVA polarizing film samples corrected by KI with different concentrations were prepared according to the above experimental steps. Figure 2 shows the comparison diagrams of PVA polarizing films with six times draw ratios (*δ*) while the concentration of KI in the correcting tank was set to 1.0%, 1.5%, 2.0%, 2.5% and 3.0% respectively. It could be seen that when the KI concentration changed from 1.0% to 2.0%, the apparent color of the PVA polarizing film obviously changed from dark blue to dark gray. When the KI concentration continued to increase up to 3.0%, the apparent color of PVA polarizing film turned slightly yellow. The color distribution of PVA polarizing film was very uniform, regardless of the apparent color transition.

**Figure 3:**
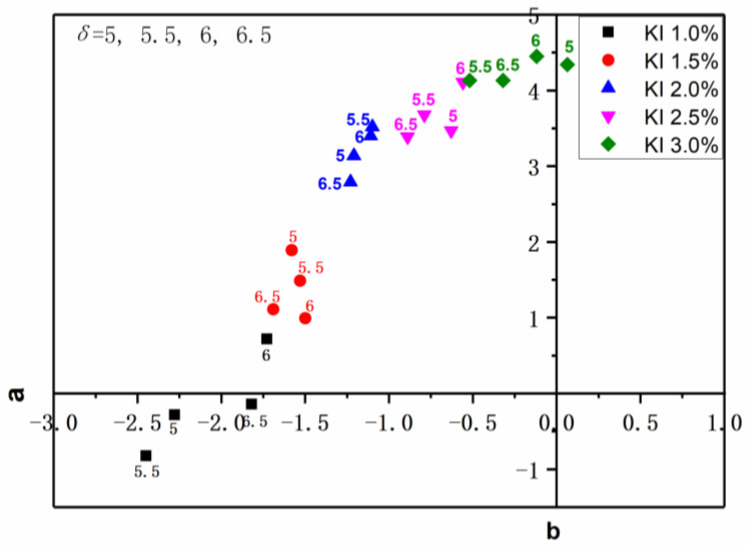
Coordinate system of chromaticity a and b values of PVA polarizing films prepared in the correcting tank with the concentration of KI being set to 1.0%, 1.5%, 2.0%, 2.5% and 3.0% respectively.

Chromaticity is one of the three optical performance indexes of the polarizer. It is usually represented by the color coordinate values *a* and *b* in the *Lab* color system of the International Lighting Commission (CIE). The *a*-value represents red and green, *+a* indicates partial red, while −*a* indicates partial green. The *b*-value represents yellow and blue, and in turn, +*b* indicates partial yellow, while −*b* indicates partial blue. For further exploration about the influence of different KI concentrations in the correcting tank on the color of PVA polarizing film, the chromaticity of PVA polarizing film was detected by the ZE6000 color difference meter of Japan Electric Color. The coordinate system diagram was composed of chromaticity *a*-value and *b*-value of PVA polarizing films, which were stretched 5, 5.5, 6, 6.5 times at 1.0%, 1.5%, 2.0%, 2.5% and 3.0% KI concentrations, respectively, as shown in Figure 3. The value of *a* increased with the increase in KI concentration in the correcting tank, and it was almost negative. When the KI concentration was greater than 1.5%, the value of *b* was positive. With the increase in KI concentration, the value of *b* gradually increased, and the color of PVA polarizing film gradually turned yellow as presented in Figure 2.

### 3.2. Characterization and Analysis of Dichroic Species in PVA Polarizing Film

**Figure 4:**
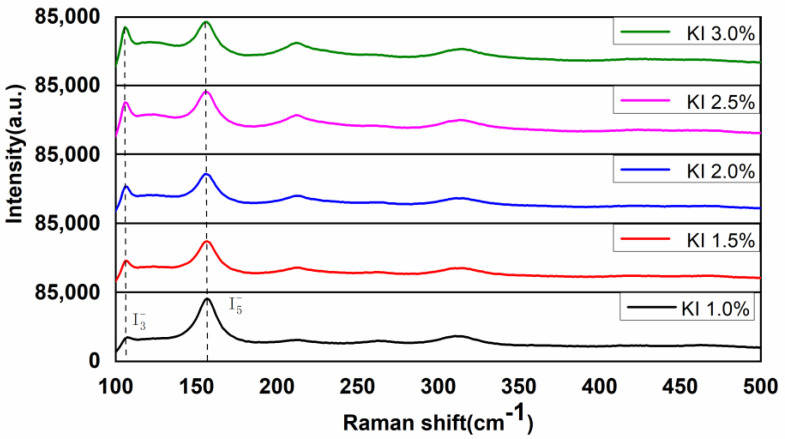
Raman spectrum of PVA polarizing films which were stretched 6 times, prepared in the correcting tank with the concentration of KI from left to right being set to 1.0%, 1.5%, 2.0%, 2.5% and 3.0% respectively.

Through the above research, the variation trend of apparent color and chromaticity *a* and *b* values of PVA polarizing film with the change of KI concentration in correcting tank had been observed. Raman test was carried out to explore the internal factors causing this change trend. According to previous research reports, the Raman vibration peak positions of $I_{3}^{-}$ and $I_{5}^{-}$ ions corresponded to about 110 cm^−1^ and 160 cm^−1^ respectively, and the relative concentrations of $I_{3}^{-}$ and $I_{5}^{-}$ ions were judged by the Raman peak intensity [48]. Figure 4 showed the Raman spectra of six times stretched PVA polarizing films prepared in 1.0%, 1.5%, 2.0%, 2.5% and 3.0% KI concentrations, respectively. It was clear that with the increase in KI concentration, the Raman vibration peak intensity at 160 cm^−1^ gradually decreased while the peak intensity at 110 cm^−1^ gradually increased. It indicated that $I_{5}^{-}$ ions in PVA polarizing film were consumed to result in the reduced concentration, but *I*_3_^−^ ions were generated to induce a gradually increased concentration.

### 3.3. Characterization and Analysis of Transmittance and Polarization

**Figure 5:**
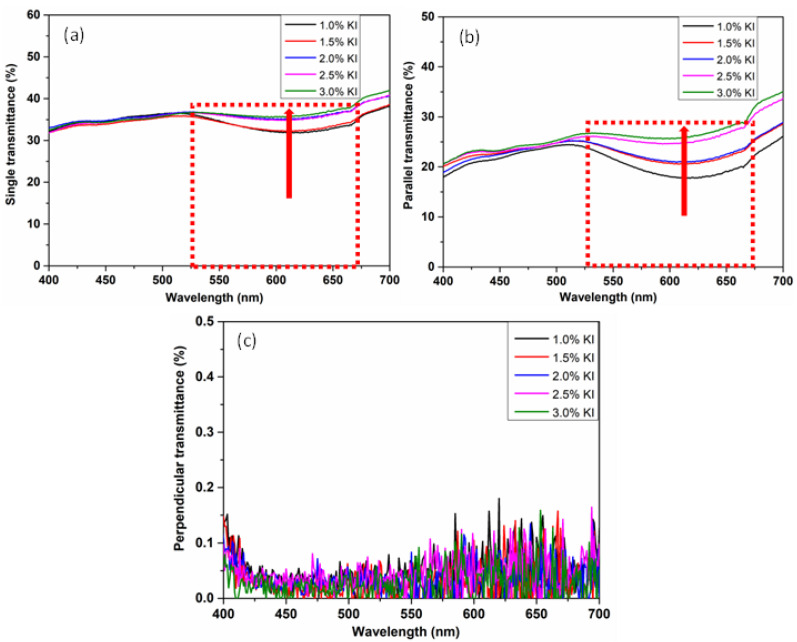
The (**a**) single, (**b**) parallel and (**c**) perpendicular transmittance spectrum of PVA polarizing films with the concentrations of KI being set to 1.0%, 1.5%, 2.0%, 2.5% and 3.0% respectively when the draw ratio was 6 all.

The ultraviolet-visible spectrophotometer (UV-Vis) was employed to detect the transmittance of the prepared PVA polarizing films. Figure 5 presented the single, parallel and perpendicular transmittance spectra of 6 times stretched PVA polarizing films prepared under 1.0%, 1.5%, 2.0%, 2.5% and 3.0% KI concentrations, respectively. From the above analysis, it was revealed that KI in the correcting tank can change the chemical equilibrium of polyiodide ions in PVA ($I_{5}^{-}+I^{-}\to 2I_{3}^{-}$) by consuming $I_{5}^{-}$ ions and generating $I_{3}^{-}$ ions. The reduction of $I_{5}^{-}$ ion concentration in PVA polarizing film can weaken its absorbance in the visible light range of around 600 nm wavelength. Therefore, the single and parallel transmittance of PVA polarizing films gradually increased in the wavelength range of 675~525 nm, as illustrated in the dotted rectangular frame of Figure 5a,b.

According to the polarizer for Thin Film Liquid Crystal Display (TFT-LCD) HG/T 4357-2012, the single transmittance (*T_s_*), parallel transmittance ($T_{∥}$) and vertical transmittance ($T_{⊥}$) of PVA polarizer can be calculated, which represents its optical average value. The degree of polarization of the polarizer is also calculated by bringing $T_{∥}$ and *T*_⊥_ into the Formula (1), which represents the comprehensive efficiency of the polarizer to produce polarized light.

(1) $$P=\sqrt{\frac{T_{∥}-T_{⊥}}{T_{∥}+T_{⊥}}}\times 100\%$$

The calculated values of single transmittance (*T_s_*), parallel transmittance($T_{∥}$), perpendicular transmittance ($T_{⊥}$) and degree of polarization (*P*) of PVA polarizing films with 6 times elongation, prepared under 1.0%, 1.5%, 2.0%, 2.5% and 3.0% KI concentrations, respectively, are listed in Table 2. It could be seen from the table that with the gradual increase in KI concentration in the correcting tank, the single transmittance, parallel transmittance and degree of polarization showed an increasing trend, and the perpendicular transmittance showed a decreasing trend. Meanwhile, the optical performance of the PVA polarizing film was improved.

## 4. Conclusions

This paper focused on the influence of KI concentration in the correcting tank on PVA polarizing films. From the results, it could be found that the KI in the correcting tank changed the chemical balance of polyiodide ions in the PVA polarizing film by consuming chromophore $I_{5}^{-}$ ions and generating $I_{3}^{-}$ ions, with the corresponding reaction equation $I_{5}^{-}+I^{-}\to 2I_{3}^{-}$. With the increase in KI concentration, more and more chromophore $I_{5}^{-}$ ions were consumed so that the apparent color of PVA polarizing films changed from dark blue to dark gray, and the chromaticity *a*-value and *b*-value increased gradually. This research achievement has important reference significance for the chromaticity adjustment of the polarizer in practical production. The absorption of $I_{3}^{-}$ and $I_{5}^{-}$ dichroic ions in PVA polarizing film in the visible range corresponds to the blue and red regions, respectively. So the decrease of $I_{5}^{-}$ ion concentration in PVA polarizing film could help improve the single and parallel transmittance of PVA polarizing film in the wavelength range of 675~525 nm, so as to improve the optical properties of PVA polarizing film.

## Figures and Tables

**Table 1:** Concentration of the solution in each tank for preparing PVA polarizing film.

Tanks	*w* (H_3_BO_3_) %	*w* (I_2_) %	*w* (KI) %	*w* (ZnCl_2_)%
Dyeing	2.80	0.11	0.92	0
Stretching	3.60	0	2.10	0
Correcting	2.50	0	1.0/1.5/2.0/2.5/3.0	0.35

**Table 2:** The calculated values of single, parallel, perpendicular transmittance and degree of polarization of PVA polarizing films with the concentrations of KI being set to 1.0%, 1.5%, 2.0%, 2.5% and 3.0%, respectively, when the draw ratio was 6.

*w* (KI)%	$T_{s}/\%$	$T_{∥}/\%$	$T_{⊥}/\%$	*P*/%
1.0	40.052	24.639	0.057	99.769
1.5	39.866	26.769	0.040	99.851
2.0	42.075	27.023	0.030	99.889
2.5	41.805	29.589	0.054	99.818
3.0	42.256	30.528	0.033	99.892

## Data Availability

The data presented in this study are available on request from the corresponding author.
